# Association Between Hyperactivity Symptoms and Somatic Complaints: Mediating and Moderating Mechanisms in Childhood Trauma and Life Events Among Chinese Male Adolescents

**DOI:** 10.3389/fpsyt.2021.630845

**Published:** 2021-09-16

**Authors:** Shuxian Wu, Tingyu Yang, Yuqiong He, Xilong Cui, Xuerong Luo, Jianbo Liu

**Affiliations:** ^1^National Clinical Research Center for Mental Disorders, Department of Psychiatry, The Second Xiangya Hospital, Central South University, Changsha, China; ^2^Hunan Key Laboratory of Psychiatry and Mental Health, China National Technology Institute on Mental Disorders, Changsha, China; ^3^Department of Child Psychiatry of Shenzhen Kangning Hospital, Shenzhen Mental Health Center, School of Mental Health, Shenzhen University, Shenzhen, China

**Keywords:** hyperactivity symptoms, trauma, life events, somatic complaints, male adolescents

## Abstract

**Background:** Prior research has found that attention deficit/hyperactivity disorder (ADHD) – particularly hyperactivity symptoms – is associated with various somatic complaints. The present study further tests the relationship between hyperactivity symptoms and somatic complaints in Chinese male adolescents and explores the underlying moderating and mediating mechanisms.

**Methods:** Our sample included 1,586 males (age = 12–16) recruited as part of an epidemiological study of child and adolescent mental disorders from April to July, 2014. Hyperactivity symptoms and somatic complaints were assessed with Achenbach's Child Behavior Checklist (CBCL), and the Childhood Trauma Questionnaire Short Form (CTQ-SF) and Adolescent Life Events Scale (ASLEC) were used to assess exposure to childhood trauma and recent life events.

**Results:** Adolescents with hyperactivity symptoms experienced more emotional abuse, physical abuse, life events, and reported more somatic complaints symptoms (*p* < 0.0083 or *p* < 0.05). Linear regression analysis showed that hyperactivity, total childhood trauma score/emotional abuse and sexual abuse and ASLEC score significantly predicted somatic complaints (all *p* < 0.05). Emotional abuse and life events mediated the relationship between hyperactivity symptoms and somatic complaints. Furthermore, childhood trauma moderated the path between hyperactivity symptoms and ASLEC in the moderation mediation model for predicting somatic complaints (*p* < 0.05).

**Conclusions:** Hyperactivity symptoms had a significant impact on somatic complaints among Chinese male adolescents. Furthermore, childhood trauma and life events affected the relationship between hyperactivity symptoms and somatic complaints. Interventions for somatic complaints in male adolescents with hyperactivity symptoms should thus consider history of childhood trauma and life events.

## Introduction

Attention deficit/hyperactivity disorder (ADHD) is a common neurodevelopmental condition characterized by inattention and/or hyperactivity-impulsivity symptoms (1). In recent years, the prevalence of ADHD has shown an increasing trend in various countries (2, 3). A systematic review and meta-analysis of 67 studies of the Chinese population found that 6.26% of children and adolescents had ADHD (4). There is a gender difference in ADHD likelihood with a significantly higher lifetime prevalence in men than in women (5). ADHD can be comorbid with various other psychiatric conditions including conduct disorder, anxiety disorder, oppositional defiant disorder, disruptive mood dysregulation disorder, and bipolar disorder (6–10). A large proportion of children and adolescents with ADHD have at least one comorbid psychiatric disorder (11). Moreover, children and adolescents with ADHD commonly have other medical disorders and physical symptoms (12). Some studies have indicated that ADHD is associated with various somatic complaints like stomachaches and migraine (13, 14). As internalizing symptoms, such as somatic complaints, are likely to be clinically ignored in children and adolescents suffering from ADHD. Therefore, it is necessary to pay more attention to the somatic complaints associated with ADHD. As a main symptom of ADHD, hyperactivity has been reported to be related to somatic complaints. For example, a 10-year study of children's psychosomatic symptoms by Santalahti et al. indicated that psychosomatic symptoms related to hyperactivity symptoms (15). Considering that boys tend to display higher levels of hyperactivity symptoms (16, 17), the purpose of this study is to further test the relationship between hyperactivity symptoms and somatic complaints in a large sample of Chinese adolescent males.

Childhood trauma refers to emotional and physical neglect as well as physical, emotional, and sexual abuse experiences during childhood and adolescence (18). There is evidence for significant associations between childhood trauma and ADHD (19–21). Several studies have shown that children with ADHD are more likely to experience traumatic events than children without ADHD (22, 23). Children with ADHD have more academic problems, such as challenges with time management and planning (24), and exhibit more impulsive behavior than typical children (25), which somewhat increases their risk of experiencing traumatic events. A systematic review of pediatric health outcomes found that experiences of childhood adverse events are associated with somatic complaints (26). Relatedly, Achenbach et al. found that the methylation level of the transient receptor potential ankyrin 1 promotor, which is related to mechanical pain sensitivity, is influenced by childhood traumatic experiences (27). Therefore, we hypothesize that childhood trauma mediates the relationship between hyperactivity symptoms and somatic complaints.

Life events refer to stressors that typically involve danger or readjustment to resume living a normal life, and thus may influence a child's development (28, 29), such as suffering from serious illness and transferring to another school. ADHD is closely related to such life events, and people who suffer from ADHD are known to experience more conflicts and adverse life events compared to people without ADHD (30, 31). Friedrichs et al. found that adults with ADHD symptoms have an increased risk of stressful life events (32). Previous studies have found that life events increase somatic complaints. For example, a study of a population cohort in the Netherlands found that negative life events predicted functional somatic symptoms (33). Similarly, a prospective study found that higher levels of negative life events at 1 year follow-up predicted higher levels of somatic complaints (34). Life events have been shown to mediate the relationship between ADHD and physical diseases. For example, Stickley et al. found that stressful life events mediated the association between ADHD symptoms and physical diseases (35). Given these findings, we predict that life events may also mediate the relationship between hyperactivity symptoms and somatic complaints.

Prior studies have found that adversities during different life periods had interactive effects on mental health. For example, Weissman et al. reported that exposure to childhood violence affected life events related to depression during the follow-up period (36). Zhong et al. found that childhood trauma changed brain function (dorsolateral pre-frontal cortex, insula, etc.) and hypothalamic-pituitary-adrenal (HPA) axis responsiveness to stress (37). Recently, Janiri et al. reported that childhood trauma increased vulnerability to stress-related psychological distress during the coronavirus disease 2019 outbreak (38). Taken together, these studies suggest that early adverse events may influence the stress response to events that occur in the future. For this reason, the present study assumes that childhood trauma may influence the mediating effect of life events on the relationship between hyperactivity symptoms and somatic complaints.

## Materials and Methods

### Participants

The data for this study are drawn from a larger epidemiological study of child and adolescent mental disorders conducted between April and July, 2014 (39). Thirteen primary and high schools (including two urban middle schools, four urban primary schools, three rural middle schools, and four rural primary schools) located in two cities in Hunan province, Central China, were selected for this study. A total of 18,778 students aged 6–16 years from the selected schools participated in the study. The participants' parents were asked to complete the Achenbach's Child Behavior Checklist (CBCL). There were 17,071 valid CBCL questionnaires. A total of 3,465 students were CBCL positive based on the results of the 17,071 valid CBCL questionnaires. The 3,465 CBCL positive participants and matched 3,465 CBCL negative participants further completed the Childhood Trauma Questionnaire Short Form (CTQ-SF) and Adolescent Life Events Scale (ASLEC). Ultimately, 3,147 students aged 12–16 years completed CTQ-SF and ASLEC. The present study only analyzed results from male students (*n* = 1,586), for who the average age was 13.57 ± 1.29 years. Among the 1,586 male students, 521 (32.8%) reported being the only child in their family, while 1,065 (67.2%) were not. Regarding environment, 442 (27.9%) lived in a city, 340 (21.4%) lived in a town, and 804 (50.7%) lived in the countryside. Regarding socio-economic status, 254 (16.02%) reported a relatively wealthy family condition, 1,237 (78%) reported an average family economic level, and the remaining 95 (6.0%) reported that their families had financial difficulties. Children and their parents (guardians) gave informed consent. Each participating student was identified by a number to maintain their anonymity. The Ethics Committee of the Second Xiangya Hospital of Central South University approved this study.

### Measures

#### Achenbach Child Behavior Checklist (CBCL)

The CBCL (40) is composed of three parts: a general situation scale, a social ability scale, and a behavioral problem scale. The behavioral problem scale comprises 113 items related to somatic complaints, hostile behavior, hyperactivity symptoms, etc. that are grouped into nine factors. Items are scored as 1, 2, or 3 according to the response (no such problem, an occasional problem, a common problem). Thus, a higher score indicates a more severe behavioral problem. The factor score is determined as the sum of all items comprising that factor. Items 49, 51, 54, 56, and 57 are related to somatic complaints. Items 1, 8, 10, 13, 17, 20, 41, 61, 62, and 64 are related to hyperactivity symptoms. The retest reliability was 0.77–0.79 and the criterion validity was 0.61–0.76 for the Chinese version of the CBCL (41).

#### Childhood Trauma Questionnaire Short Form (CTQ-SF)

The CTQ-SF is composed of 28 items that form five subscales, namely emotional abuse, physical abuse, emotional neglect, physical neglect, and sexual abuse, and three validity items (18). Emotional and physical neglect is defined as inaction during the care process that results in potential or actual harm, e.g., when emotional needs are ignored by caregivers (42). Physical abuse refers to direct beating, burns, and biting, while sexual abuse refers to cases where children and adolescents participate in sexual activities that they do not fully understand, that they cannot consent to, or that violate family roles (42). Emotional abuse refers to threatening behavior, refusal and hostile verbal abuse, or exploitation (43). The questionnaire is suitable for children and adolescents aged 12–16 (44). Participants responded to each item with a score of 1, 2, 3, 4, or 5 corresponding to the frequency they experienced the specific item (never, occasionally, sometimes, often, or always). Based on previous work, we defined an emotional abuse score ≥ 13 as moderate to severe emotional abuse, a physical abuse score ≥ 10 as moderate to severe physical abuse, a sexual abuse score ≥ 8 as moderate to severe sexual abuse, an emotional neglect score ≥ 15 as moderate to severe emotional neglect, and a physical neglect score ≥ 10 points as moderate to severe physical neglect (45). If a participant experienced any kind of moderate to severe abuse or neglect, they were considered to have experienced childhood trauma. The Chinese version of the CTQ-SF has good reliability and validity, and the Cronbach α coefficient was 0.77 (46).

#### Adolescent Life Events Scale (ASLEC)

The ASLEC is a 27-item scale developed in 1987 by Liu et al. based on the physiological and psychological characteristics of Chinese adolescents (47). Each item relates to a common stressor experienced by adolescents over the past 12 months and the occurrence and impact of adolescent life events are evaluated. If life events occurred, items are rated on a five-point scale based on the psychological experience of the event. A higher ASLEC score indicates greater stress reaction. Due to its simplicity, the ASLEC is widely used in China (48). The Cronbach α coefficient was 0.91 and the comparative fit index was 0.9 (49).

### Statistical Analysis

Data were analyzed using SPSS version 21 statistical software (IBM). Independent sample *t*-tests were used to compare scores of different types of abuse /total childhood trauma, somatic complaints, and ASLEC scores between the hyperactive and non-hyperactive groups. To avoid the risk of false positive results when comparing the scores of different types of abuse/total childhood trauma, Bonferroni correction was applied for multiple testing. Relationships among hyperactivity scores, ASLEC scores, somatic complaints, and CTQ-SF scores were assessed using Pearson correlation analysis. Hyperactivity scores, ASLEC scores, and CTQ-SF dimension scores were used to predict somatic complaints via linear regression. The Process 3.2 plug-in was used for the mediation analysis (selected model 4) and moderation mediation analysis (selected model 58). The mediation analysis used the bootstrapping method with 5,000 iterations. A 95% confidence interval of the bootstrapping method that did not include 0 was considered to indicate a statistically significant difference. In line with Bonferroni correction for multiple comparison testing for the different types of abuse/total childhood trauma, *p* < 0.0083 (0.05/6) was selected as the level of statistical significance. For other analyses, *p* < 0.05 was selected as the level of statistical significance.

## Results

### Comparison of Somatic Complaints and Childhood Trauma Scores (All Dimensions and Total Score) Between the Hyperactive Group and Non-hyperactive Group

Emotional abuse and physical abuse scores for the hyperactive group were significantly higher than those for the non-hyperactive group (all *p* < 0.0083). The hyperactive group also had significantly higher ASLEC and somatic complaints scores than the non-hyperactive group (both *p* < 0.05; Table 1).

**Table 1:** Comparison of various childhood abuse scores, total ASLEC score, and somatic complaints between the hyperactive and non-hyperactive groups.

**Variables**		***N***	**Mean ± Standard deviation**	***T***	***p***
Total childhood trauma score	Non-hyperactive	1,481	39.28 ± 10.46	−1.870	0.062
	Hyperactive	105	41.28 ± 12.29		
Emotional abuse	Non-hyperactive	1,481	6.58 ± 2.50	−3.680	<0.001
	Hyperactive	105	7.53 ± 3.50		
Physical abuse	Non-hyperactive	1,481	6.06 ± 2.38	−3.159	0.002
	Hyperactive	105	6.84 ± 3.04		
Sexual abuse	Non-hyperactive	1,481	5.52 ± 1.97	−0.191	0.849
	Hyperactive	105	5.56 ± 1.29		
Emotional neglect	Non-hyperactive	1,481	11.37 ± 5.13	−1.606	0.108
	Hyperactive	105	12.21 ± 5.54		
Physical neglect	Non-hyperactive	1,481	9.74 ± 3.37	1.772	0.077
	Hyperactive	105	9.13 ± 3.49		
ASLEC	Non-hyperactive	1,481	15.69 ± 15.48	−6.136	<0.001
	Hyperactive	105	25.27 ± 14.98		
Somatic complaints	Non-hyperactive	1,481	2.96 ± 4.17	−13.532	<0.001
	Hyperactive	105	8.76 ± 5.23		

*ASLEC, Total adolescent life events scale score*.

### Correlation Analysis of All Relevant Scores

As shown in Table 2, hyperactivity score was positively related to scores for emotional abuse, physical abuse, sexual abuse, emotional neglect, ASLEC, and somatic complaints (*r* = 0.088–0.534, all *p* < 0.01). Hyperactivity score was negatively related to physical neglect score (*r* = −0.049, *p* < 0.05). Somatic complaints score was positively related to scores for emotional abuse, physical abuse, sexual abuse, emotional neglect, and ASLEC (*r* = 0.096–0.255, all *p* < 0.01). The total childhood trauma score was positively related to scores for hyperactivity, emotional abuse, physical abuse, sexual abuse, emotional neglect, physical neglect, ASLEC, and somatic complaints (*r* = 0.136–0.767, all *p* < 0.01).

**Table 2:** Correlations among scores for hyperactivity, childhood trauma, ASLEC, and somatic complaints (*n* = 1,586).

	**Hyperactivity**	**Total childhood trauma score**	**Emotional abuse**	**Physical abuse**	**Sexual abuse**	**Emotional neglect**	**Physical neglect**	**ASLEC**	**Somatic complaints**
Hyperactivity	1								
Total childhoodtrauma score	0.136**	1							
Emotional abuse	0.198**	0.678**	1						
Physical abuse	0.152**	0.654**	0.640**	1					
Sexual abuse	0.088**	0.560**	0.467**	0.477**	1				
Emotional neglect	0.108**	0.767**	0.272**	0.240**	0.169**	1			
Physical neglect	−0.049*	0.655**	0.219**	0.200**	0.225**	0.402**	1		
ASLEC	0.281**	0.220**	0.356**	0.307**	0.157**	0.127**	−0.088**	1	
Somatic complaints	0.534**	0.185**	0.255**	0.180**	0.163**	0.096**	0.015	0.242**	1
Mean	4.48	39.41	6.64	6.11	5.53	11.43	9.70	16.33	3.34
Standard deviation	3.43	10.60	2.58	2.44	1.94	5.16	3.38	15.63	4.49

*ASLEC, Total adolescent life events scale score;*

* *p < 0.05*,

** *p < 0.01*.

### Linear Regression Model for Predicting Somatic Complaints

The results of linear regression analysis including hyperactivity, different types of childhood trauma, and life events showed that hyperactivity score (β = 0.492, *p* < 0.001), emotional abuse score (β = 0.119, *p* < 0.001), sexual abuse score (β = 0.062, *p* = 0.011), and ASLEC score (β = 0.06, *p* = 0.011) significantly predicted somatic complaints (Table 3A).

**Table 3A:** Linear regression analysis for hyperactivity, different types of childhood trauma, and life events predicting somatic complaints.

**Model**	**Variables**	**USC**		**SC**	***t***	***P***
		**B**	**SE**	**β**		
1	Hyperactivity	0.697	0.028	0.534	25.108	<0.001
2	Hyperactivity	0.658	0.028	0.503	23.373	<0.001
	Emotional abuse	0.233	0.05	0.134	4.69	<0.001
	Physical abuse	−0.02	0.052	−0.011	−0.384	0.701
	Sexual abuse	0.142	0.057	0.061	2.485	0.013
	Emotional neglect	−0.002	0.02	−0.002	−0.105	0.916
	Physical neglect	0.001	0.031	0	0.017	0.987
3	Hyperactivity	0.643	0.029	0.492	22.394	<0.001
	Emotional abuse	0.206	0.051	0.119	4.07	<0.001
	Physical abuse	−0.036	0.052	−0.019	−0.679	0.497
	Sexual abuse	0.145	0.057	0.062	2.532	0.011
	Emotional neglect	−0.006	0.02	−0.007	−0.317	0.751
	Physical neglect	0.016	0.032	0.012	0.5	0.617
	ASLEC	0.017	0.007	0.06	2.552	0.011

*SC, Standardized coefficient; USC, Unstandardized coefficient; SE: Standard error*.

The results of linear regression analysis including hyperactivity, severity of childhood trauma (total childhood trauma score), and life events showed that hyperactivity score (β = 0.497*, p* < 0.001), total score of childhood trauma (β = 0.1, *p* < 0.001), and ASLEC score (β = 0.081, *p* < 0.001) significantly predicted somatic complaints (Table 3B).

**Table 3B:** Linear regression analysis for hyperactivity, severity of childhood trauma, and life events predicting somatic complaints.

**Model**	**Variables**	**USC**		**SC**	***t***	***P***
		**B**	**SE**	**β**		
1	Hyperactive	0.697	0.028	0.534	25.108	<0.001
2	Hyperactive	0.677	0.028	0.518	24.358	<0.001
	Total childhood trauma score	0.048	0.009	0.115	5.385	<0.001
3	Hyperactive	0.65	0.029	0.497	22.674	<0.001
	Total childhood trauma score	0.042	0.009	0.1	4.613	<0.001
	ASLEC	0.023	0.006	0.081	3.625	<0.001

*SC, Standardized coefficient; USC, Unstandardized coefficient; SE: Standard error*.

### Mediation Analysis for the Relationship Between Hyperactivity and Somatic Complaints

Based on the hypotheses described earlier, model 4 of the Process 3.2 plug-in was used to further examine the mediating effects of childhood trauma and life events on the relationship between somatic complaints and hyperactivity. The results showed that life events and emotional abuse mediated the relationship between hyperactivity and somatic complaints (the 95% confidence interval of bootstrapping did not include 0) in the mediation model; however, the severity of childhood trauma (total childhood trauma score) did not mediate the relationship between hyperactivity and somatic complaints (the 95% confidence interval of bootstrapping included 0) in the mediation model (Tables 4A,B).

**Table 4A:** Test of the indirect path for different types of childhood trauma and life events mediating the relationship between hyperactivity and somatic complaints.

**Paths**	**Effect**	**Boot SE**	**Boot LLCL**	**Boot ULCL**
Hyperactivity → ASLEC → Somatic complaints	0.022	0.0107	0.0014	0.0439
Hyperactivity → EA → Somatic complaints	0.0308	0.0106	0.011	0.0522
Hyperactivity → PA → Somatic complaints	−0.0038	0.008	−0.0207	0.0109
Hyperactivity → SA → Somatic complaints	0.0072	0.0063	−0.0049	0.0193
Hyperactivity → EN → Somatic complaints	−0.0011	0.0028	−0.0068	0.0046
Hyperactivity → PN → Somatic complaints	−0.0008	0.0017	−0.0048	0.0023

*EA, Emotional abuse; PA, Physical abuse; SA, Sexual abuse; EN, Emotional neglect; PN, Physical neglect; ASLEC, Total adolescent life events scale score; SE: Standard error; Boot, Bootstrapping; LLCL: The lower limit of the 95% confidence interval; ULCL: The upper limit of the 95% confidence interval*.

**Table 4B:** Test of the indirect path for the severity of childhood trauma and life events mediating the relationship between hyperactivity and somatic complaints.

**Paths**	**Effect**	**Boot SE**	**Boot LLCL**	**Boot ULCL**
Hyperactivity → ASLEC → Somatic complaints	0.0296	0.0096	0.0118	0.0495
Hyperactivity → Total childhood trauma score → Somatic complaints	0.0177	0.0112	−0.0001	0.0421

*ASLEC, Total adolescent life events scale score; SE, Standard error; Boot, Bootstrapping; LLCL: The lower limit of the 95% confidence interval; ULCL: The upper limit of the 95% confidence interval*.

### Moderation Mediation Analysis for the Relationship Between Hyperactivity and Somatic Complaints

If a participant experienced any type of moderate to severe abuse or neglect, they were considered to have experienced childhood trauma. Based on our hypotheses, model 58 of the Process 3.2 plug-in was used to examine whether childhood trauma moderated two paths (hyperactivity → life events and life events → somatic complaints) in the mediation model. In the moderated mediation model, hyperactivity was treated as an independent variable, somatic complaints were treated as the dependent variable, life events were treated as a mediator, and the presence/absence of childhood trauma was treated as a moderator.

**Figure 1:**
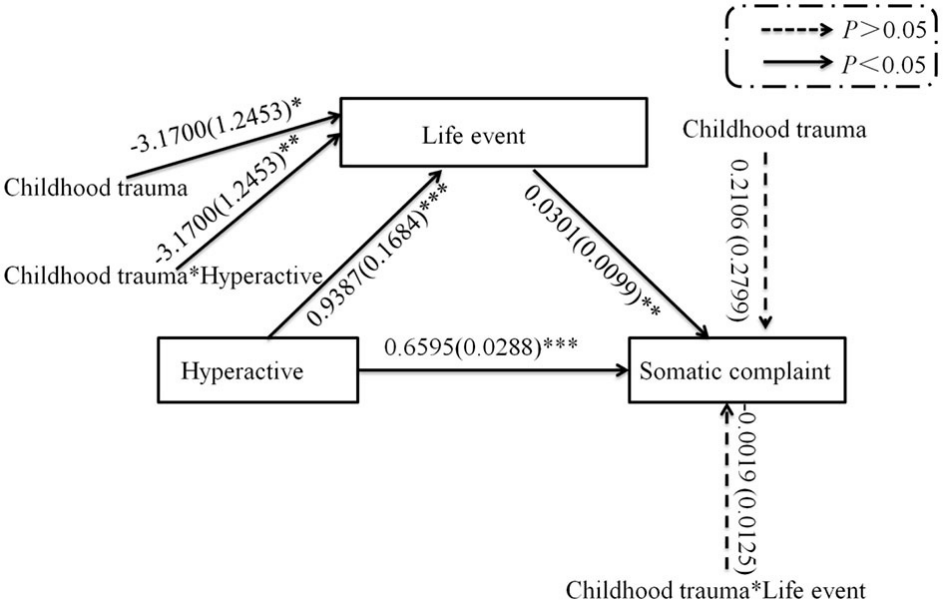
Path of moderation mediation analysis for the relationship between hyperactivity and somatic complaints. *p < 0.05, **p < 0.01, ***p < 0.001.

**Figure 2:**
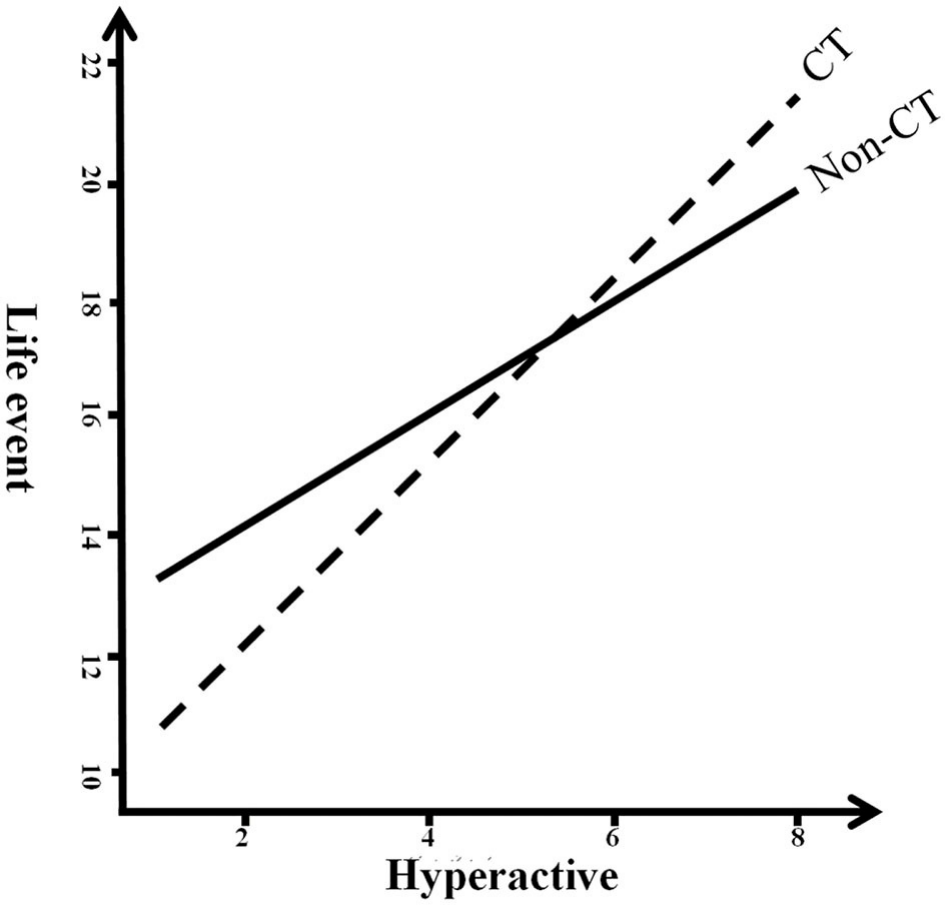
Childhood trauma moderated the relationship between hyperactivity and life events in moderation mediation analysis. CT, Childhood trauma.

The results showed that childhood trauma moderated the path between hyperactivity and life events (*p* = 0.0076; see Figures 1, 2); however, childhood trauma did not moderate the path between life events and somatic complaints (*p* = 0.8802; see Figure 1) in the mediation analysis of the relationship between hyperactivity and somatic complaints. Furthermore, life events mediated the relationship between hyperactivity and somatic complaints in persons with/without childhood trauma (the 95% confidence interval of bootstrapping did not include 0; Table 5).

**Table 5:** Test of the indirect path for the relationship between hyperactivity and somatic complaints in exposed and non-exposed childhood trauma populations in moderation mediation analysis.

**Childhood trauma**	**Paths**	**Effect**	**Boot SE/SE**	**Boot LLCL**	**Boot ULCL**
Yes	Hyperactivity → ASLEC → Somatic complaints	0.0432	00146	0.0168	0.0737
No	Hyperactivity → ASLEC → Somatic complaints	0.0282	0.0102	0.0109	0.0507

*ASLEC, Total adolescent life events scale score; SE, Standard error; Boot, Bootstrapping; LLCL: The lower limit of the 95% confidence interval; ULCL: The upper limit of the 95% confidence interval*.

## Discussion

The present study is a cross-sectional study to explore the relationship between hyperactivity symptoms and somatic complaints, and the first to explore the mediating and moderating influences of recent life events and childhood trauma, in male adolescents. We found that, compared to non-hyperactive male adolescents, hyperactive male adolescents suffered more emotional abuse, physical abuse, and life events. Previous studies have found similar results in children with ADHD (50, 51). For instance, children with ADHD have higher exposure to childhood trauma than those without ADHD (52). Correlational analysis revealed that hyperactivity symptoms were associated with life events and all dimensions of childhood trauma. Hyperactive teenagers are more likely to be criticized, bullied, or injured (53), this might because they tend to violate social norms and often exhibit impulsive behavior. Additionally, parental or teacher criticism is more commonly accompanied by physical violence and verbal abuse than sexual abuse in China (54). This may partly explain the results that hyperactive male adolescents experienced more emotional and physical abuse than non-hyperactive male adolescents, while experiencing no significant difference in sexual abuse and neglect.

There is evidence that adolescents with ADHD have significantly higher somatic complaints scores than control adolescents (55, 56). Similar to previous research findings, the present study found that adolescents who screened positively for hyperactivity problems had more somatic complaints than those who screened negatively. Furthermore, correlation analysis found that somatic complaints were significantly correlated with hyperactivity symptoms as well as with childhood trauma and life events. Among adults in the general population, somatic complaints are strongly associated with reporting of past negative events (e.g., loss and abuse) (57). As expected, and consistent with previous studies (26, 58), the current study found that, among male adolescents, exposure to childhood maltreatment or adversity was correlated with somatic complaints. Notably, physical neglect was not significantly correlated with somatic complaints. It is possible that parental neglect of children's physical needs can lead to children ignoring physical discomfort or rarely complaining about physical discomfort, as such complains do not receive their parents' attention.

Previous studies of children and adolescents have found that ADHD symptoms are significantly related to somatic complaints (59) and that migraines and recurrent abdominal pain are more common in ADHD adolescents than healthy control (14). The linear regression found that hyperactivity symptoms are positively associated with somatic complaints in male adolescents. Moreover, life events, as well as childhood trauma, were found to be an important factor for somatic complaints. Growing evidence indicates that childhood trauma is associated with a significantly increased risk of medical disorders (60) and somatic complaints in adults (61, 62), that childhood adversity is associated with somatic complaints in adolescents (26), and that childhood maltreatment scores predict somatic complaints (63, 64). Our linear regression analysis further showed that emotional abuse and sexual abuse positively predicted somatic complaints, while physical abuse, emotional neglect, and physical neglect did not. Although the results for physical abuse, emotional neglect, and physical neglect showed no predictive effect in the statistical analysis, this does not mean that they have no predictive effect on somatic complaints. For instance, Ibeziako et al. found that neglect affected the range of pain sites in pediatric patients with somatic symptoms and related disorders (65). Longitudinal research is needed to verify this relationship. For boys with somatic complaints, it is necessary to teach them how to effectively cope with the adverse effects of childhood trauma, especially emotional and sexual abuse, and to cope with the challenges of hyperactivity symptoms and life events. It is well-known that stress spikes during adolescence (66). Teens who vent their emotions to close peers but do not receive supportive responses have more somatic complaints (67). Somatic complaints may be an adolescent means of coping with stress or negative emotions due to recent adversities.

The results of our mediation analysis firstly showed only emotional abuse and life events mediated the relationship between hyperactivity symptoms and somatic complaints, whereas emotional neglect, sexual abuse, physical neglect, and physical abuse did not. Moreover, total scores of childhood trauma did not have a mediating role in the analysis. This means that the effect of hyperactivity symptoms on somatic complaints is partly through emotional abuse and life events. Among male adolescents who have experienced recent life events or emotional abuse, hyperactivity symptoms may lead to more problematic somatic complaints. The reason for this pattern may be that emotional abuse in the CTQ includes blatant belittling and humiliation, and male adolescents with hyperactivity symptoms are more likely to be criticized and to experience shame for their disorderly behavior. Shame is a mechanism through which emotional abuse can lead to somatic complaints (61). Notably, Rajindrajith et al. found that emotional abuse was significantly associated with constipation in children, and that children with constipation with a history of emotional abuse had a higher somatization index compared to those with a history of sexual abuse and physical abuse (68). Emotional abuse may have a greater impact on children's somatic complaints than other forms of childhood trauma. Currently, there is no evidence of a relationship between physical abuse or physical neglect and somatic complaints (69). Considering that a previous study found a synergistic effect of childhood trauma and life events on behavioral problems (70), interventions relevant to both childhood trauma and life events are needed to reduce somatic complaints of male adolescents with hyperactivity symptoms. Our results also suggest that male adolescents with ADHD who have experienced emotional abuse may present with more somatic complaints. For such ADHD adolescents, psychotherapy could focus on childhood trauma (especially emotional abuse) and life events, and clinical treatment may need to focus not just on somatic complaints, but also on the impact of childhood trauma and life events. Reducing the occurrence of emotional abuse and life events, and improving the environment of adolescents are of great importance for the health of adolescents.

Lastly, this study found a moderating role of childhood trauma in the mediation model for hyperactivity symptoms → life events. This finding is consistent with our hypothesis that male adolescents with hyperactivity symptoms and a history of childhood trauma exposure would have more somatic complaints when experiencing recent life events. However, the mechanism for this relationship is unclear. It could be linked to post-traumatic changes in brain function or HPA axis responsiveness to stress (37). Based on the findings described above, interventions to address somatic complaints in male adolescents with hyperactivity symptoms should consider the effects of trauma and life events alone, and the interaction between childhood traumatic experiences and life events should be addressed.

### Limitations

This study is subject to several limitations. First, as this is a cross-sectional study with recall bias, further study is needed to explore causal relationships. Second, this study only examined the influence of childhood trauma and life events on the relationship between hyperactivity symptoms and somatic complaints in the general population. For applications in ADHD, this relationship needs to be tested in ADHD patients. Third, there may be some overlap between childhood trauma and life events that occur in adolescence, which may impact the results of this study. Fourth, the childhood trauma measure used in this study only investigated severity, and did not consider age at the time of trauma or frequency of trauma, which may affect the results. Fifth, it is well-known that self-evaluation is suitable for assessing internalizing behaviors; however, for externalizing behaviors (like hyperactivity), external evaluation is better. This may affect the validity or reliability of the results.

## Conclusion

Hyperactivity symptoms, childhood trauma, and life events were all found to impact somatic complaints among male adolescents. This study firstly found that emotional abuse and life events mediated the relationship between hyperactivity symptoms and somatic complaints, and that childhood trauma had moderating effects on the path between hyperactivity symptoms and life events in the mediation model for predicting somatic complaints. In light of these findings, interventions to address somatic complaints among male adolescents with hyperactivity symptoms should consider the impact of childhood trauma and life events.

## Data Availability Statement

The original contributions presented in the study are included in the article, further inquiries can be directed to the corresponding authors.

## Ethics Statement

The studies involving human participants were reviewed and approved by The Ethics Committee of the Second Xiangya Hospital of Central South University. Children and their parents (guardians) gave informed consent.

## Author Contributions

SW and TY was responsible for writing the manuscript. YH was responsible for data processing and writing the manuscript. XC participated in the design, investigation, and evaluation of the study. XL participated in the design, investigation, and evaluation of the study and contributed to critical revision. JL participated in the investigation and contributed to critical revision. All authors have read and approved the manuscript.

## Funding

This study was supported by the National Science and Technology Support Plan - Epidemiological Investigation of Mental Disorders among Chinese Children and Adolescents (Grant No. 2012BAI01B02), Science and Technology Innovation Committee of Shenzhen (JCYJ20190809155019338), Guangdong Basic and Applied Basic Research Foundation (2019A1515110047), and Hunan Provincial Innovation Foundation for Postgraduates (Grant No. CX2019159).

## Conflict of Interest

The authors declare that the research was conducted in the absence of any commercial or financial relationships that could be construed as a potential conflict of interest.

## Publisher's Note

All claims expressed in this article are solely those of the authors and do not necessarily represent those of their affiliated organizations, or those of the publisher, the editors and the reviewers. Any product that may be evaluated in this article, or claim that may be made by its manufacturer, is not guaranteed or endorsed by the publisher.
